# Understanding psychosocial determinants of malaria behaviours in low-transmission settings: a scoping review

**DOI:** 10.1186/s12936-023-04831-9

**Published:** 2024-01-10

**Authors:** Albert Casella, April Monroe, Michael Toso, Gabrielle Hunter, Carol Underwood, Ruchita Pillai, Jayme Hughes, Lynn M. Van Lith, Shelby Cash, Jimee Hwang, Stella Babalola

**Affiliations:** 1 Breakthrough ACTION Project, Johns Hopkins Center for Communication Programs, 111 Market Place, Suite 310, Baltimore, MD 21202 USA; 2 Department of Health, Behavior, & Society, Johns Hopkins Bloomberg School of Public Health, Baltimore, USA; 3 Department of International Health, Johns Hopkins Bloomberg School of Public Health, Baltimore, USA; 4 U.S. President’s Malaria Initiative, Malaria Branch, US Centers for Disease Control and Prevention, Atlanta, GA USA

**Keywords:** Malaria, Elimination, Low-transmission, Ideation, Communication, ITN, Knowledge, Attitudes, Prevention

## Abstract

**Background:**

Recent estimates show progress toward malaria elimination is slowing in many settings, underscoring the need for tailored approaches to fight the disease. In addition to essential structural changes, human behaviour plays an important role in elimination. Engagement in malaria behaviours depends in part on psychosocial determinants such as knowledge, perceived risk, and community norms. Understanding the state of research on psychosocial determinants in low malaria transmission settings is important to augment social and behaviour change practice. This review synthesizes research on psychosocial factors and malaria behaviours in low-transmission settings.

**Methods:**

A systematic search of peer-reviewed literature and supplemental manual search of grey literature was conducted using key terms and eligibility criteria defined a priori. Publications from 2000–2020 in the English language were identified, screened, and analysed using inductive methods to determine the relationship between the measured psychosocial factors and malaria behaviours.

**Results:**

Screening of 961 publications yielded 96 for inclusion. Nineteen articles collected data among subpopulations that are at increased risk of malaria exposure in low-transmission settings. Purposive and cluster randomized sampling were common sampling approaches. Quantitative, qualitative, and mixed-methods study designs were used. Knowledge, attitudes, and perceived risk were commonly measured psychosocial factors. Perceived response-efficacy, perceived self-efficacy, and community norms were rarely measured. Results indicate positive associations between malaria knowledge and attitudes, and preventive and care-seeking behaviour. Studies generally report high rates of correct knowledge, although it is comparatively lower among studies of high-risk groups. There does not appear to be sufficient extant evidence to determine the relationship between other psychosocial variables and behaviour.

**Conclusions:**

The review highlights the need to deploy more consistent, comprehensive measures of psychosocial factors and the importance of reaching subpopulations at higher risk of transmission in low transmission contexts. Malaria-related knowledge is generally high, even in settings of low transmission. Programmes and research should work to better understand the psychosocial factors that have been positively associated with prevention and care-seeking behaviours, such as norms, perceived response efficacy, perceived self-efficacy, and interpersonal communication. These factors are not necessarily distinct from that which research has shown are important in settings of high malaria transmission. However, the importance of each factor and application to malaria behaviour change programming in low-transmission settings is an area in need of further research. Existing instruments and approaches are available to support more systematic collection of psychosocial determinants and improved sampling approaches and should be applied more widely. Finally, while human behaviour is critical, health systems strengthening, and structural interventions are essential to achieve malaria elimination goals.

**Supplementary Information:**

The online version contains supplementary material available at 10.1186/s12936-023-04831-9.

## Background

Despite significant gains over the past two decades, malaria continues to take the lives of almost 1700 people each day [1]. The most recent World Health Organization (WHO) estimates show progress has stalled in recent years, underscoring the importance of addressing gaps in access to and use of core malaria interventions, and the need for innovative and more tailored approaches to fighting the disease [1].

Human behaviour plays a fundamental role in the prevention, diagnosis, and treatment of malaria. The effectiveness and longevity of insecticide-treated nets (ITNs) have been shown to depend on levels of use and appropriate care of available nets [2]. Likewise, indoor residual spraying (IRS) effectiveness depends on factors such as acceptance of sprayers in the home, willingness to remove household items during spraying, and refraining from post-spray wall modification [3]. Efficacious treatment relies on prompt care-seeking [4] and taking the full course of medication as prescribed to be effective [5].

Whether someone engages in these behaviours may depend on factors such as their level of knowledge about malaria, attitudes towards the recommended solutions, the extent to which malaria is viewed as a threat, perceptions about how well an intervention works (i.e., perceived response-efficacy), their perceived self-efficacy to use it, and social norms around prevention and care-seeking behaviours. Understanding which specific combination of factors influence malaria behaviours in a given context is essential to promoting effective social and behaviour change (SBC) strategies to increase the impact of interventions.

**Fig. 1:**
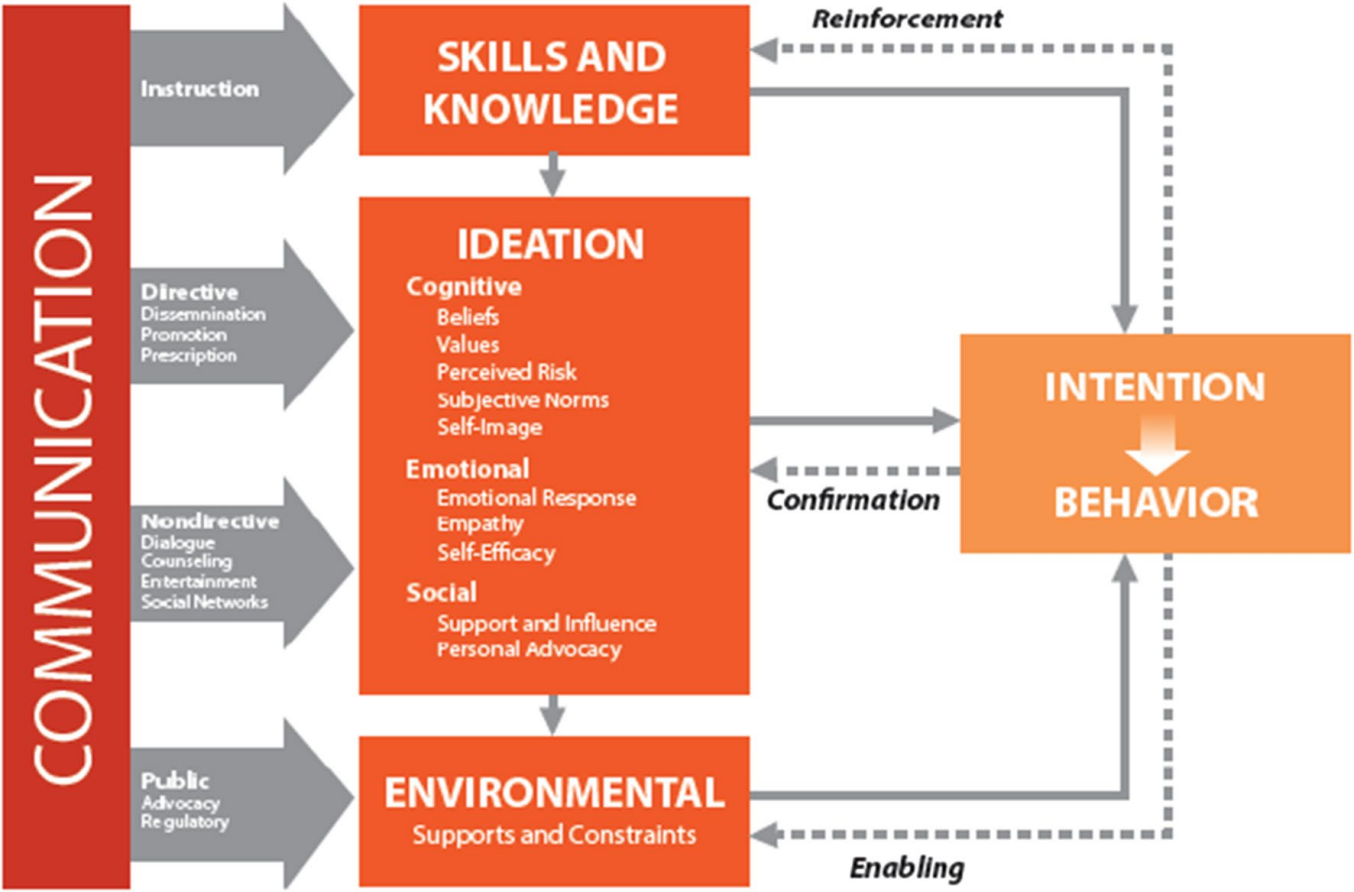
Ideation model of communication

The ideation model (Fig. 1), which encompasses a broad set of psychosocial determinants of behaviour [6], has been used to understand and successfully promote a range of health behaviours across HIV treatment and prevention [7, 8], family planning [9–12], water, sanitation and hygiene [13], and the Ebola response [14].

A growing body of literature describes how cognitive, emotional, and social constructs contained in the ideation model (i.e., ideational factors) are associated with individual and household malaria behaviours. Single and multi-country studies conducted throughout sub-Saharan Africa have, for example, found ideational variables to be predictive of ownership [15], use [16–18], and care of insecticide-treated mosquito nets (ITNs). Studies have also found ideational variables linked to both care-seeking for fever [19, 20] and uptake of intermittent preventive treatment of malaria during pregnancy (IPTp) [21].

While research on ideational factors has been used to better understand and promote malaria behaviours in high-transmission settings, less is known about how ideational factors present in low-transmission settings and the role of these factors in malaria-related behaviours. Areas with low and very low transmission (defined further in *Methods* section) are distinct from areas with moderate to high levels of transmission in several important ways including: increased geographic focalization of malaria cases; a shift toward cases among adults, particularly men; increased importance of outdoor and occupation-based exposure; and imported malaria cases [22].

While core malaria interventions (ITNs, IRS, testing and treatment for malaria, and IPTp) remain important, a number of other interventions, which may or may not be WHO-recommended, are implemented by malaria programmes in lower transmission settings to target malaria parasites and vectors [23]. These include the use of personal protection measures, such as repellents or insecticide-treated hammock nets to protect higher risk groups, larval source management (LSM), active case detection (ACD) to identify new cases within a household or community, and presumptive chemoprevention approaches, such as mass drug administration (MDA), targeted drug administration, and reactive drug administration to eliminate the parasite reservoir [23].

Understanding specific behaviours related to malaria interventions in low-transmission settings, the drivers of those behaviours, and the context in which they occur will play an important role in achieving malaria elimination [24]. Interventions to affect behaviour change at the individual and household level, such as consistent ITN use will likely be most effective alongside structural changes such as expanding improved housing and LSM [25]. This review was carried out to identify existing research on ideational factors and malaria behaviours in low-transmission contexts and gaps in evidence that can be filled through future research.

## Methods

### Search strategy

A systematic search of eight academic databases (PubMed, Public Health ProQuest, Academic Search Ultimate, Web of Science, SOC Index, Communication & Mass Media, EMBASE, and Science Direct) was conducted using key terms defined a priori (see Additional file 1). An additional manual search of reference lists of publications belonging to the University of California San Francisco Malaria Elimination Initiative (UCSF-MEI), the Roll Back Malaria (RBM) Partnership to End Malaria, and The U.S. President’s Malaria Initiative (PMI) was performed. For the purposes of this review, “low-transmission settings” were defined as a context characterized by annual parasite incidence (API) lower than 250 cases per 1000 population or *Plasmodium falciparum*/*Plasmodium vivax* prevalence rate of < 10%. This definition aligns with *A Framework for Malaria Elimination* of the WHO [24]*,* which also produced a list of countries that qualify as low-transmission settings according to this definition. This list of countries served as an initial list to inform the search strategy, although searches were not limited only to those identified in the report.

Key search terms included a combination of: “malaria”, one or more psychosocial constructs within the ideational model; and either one or more geographic terms that reflect a low-transmission setting or one or more terms that reflect a key subpopulation that evidence suggests may experience risk focalization in low-transmission settings (see Additional file 1). These key subpopulations include, among others, seasonal workers, forest goers, miners, migrant workers, and internally displaced persons (IDPs). Specifically, psychosocial constructs included in searches included: malaria knowledge; attitudes toward malaria; perceived risk of malaria; perceived response-efficacy of malaria prevention and treatment approaches; one’s perceived self-efficacy to take certain malaria actions; and interpersonal communication. Additionally, three types of perceived social norms were included in the search: the *perceived descriptive norm* (referring to how an individual perceives their community to think, feel, or act – in this case, related to malaria behaviours); the *perceived subjective norm* (one’s perceptions of what others who are important to the individual want them to do); and the *perceived injunctive norm* (whether an individual perceives most people in the community to approve of an action).

### Eligibility criteria

The relevance of publications was determined based on inclusion and exclusion criteria defined in Table 1. To minimize outdated information, only studies published from 2000 to 2020 were included. No restrictions were placed on the study design. Publications were limited to the English language. Publications were included if the focus of the research was on investigating the relationship between individual or community psychosocial determinants and malaria behaviour.

**Table 1:** Inclusion and exclusion criteria

Criterion	Inclusion	Exclusion
Language	Published in English	Not published in English
Study focus	Investigating behavioral determinants (as defined by ideation model) of malaria prevention/treatment behaviors	Does not include investigation into behavioral determinants
Data collection setting	Within at least one of or any combination of low-transmission countries or regions as defined by WHO’s *A Framework for Malaria Elimination* [24] If the title or abstract of the article included a key term referring to the setting as a “low-transmission area/zone/country/setting”, the article was also included and verification using API or *P. falciparum*/*P*. *vivax* prevalence conducted during data extraction	Any country or setting not belonging in a low-transmission setting

### Study selection

Search results were imported into Endnote version X9. Following the removal of duplicate articles, study titles were screened to assess eligibility. Then, a separate full-text screening of retained articles was conducted to confirm eligibility. Results were excluded if the publication did not meet all inclusion criteria. Reviewers discussed and resolved through consensus any discordances during the selection process.

### Results synthesis

Key characteristics of publications that met inclusion criteria were summarized, including focus population of the study, study design and sampling method, measurement of each psychosocial construct (cognitive, social, emotional) contained in the ideation model, and the malaria behaviour of focus. Following the charting of publications, an inductive thematic analysis method identified common themes related to findings.

## Results

**Fig. 2:**
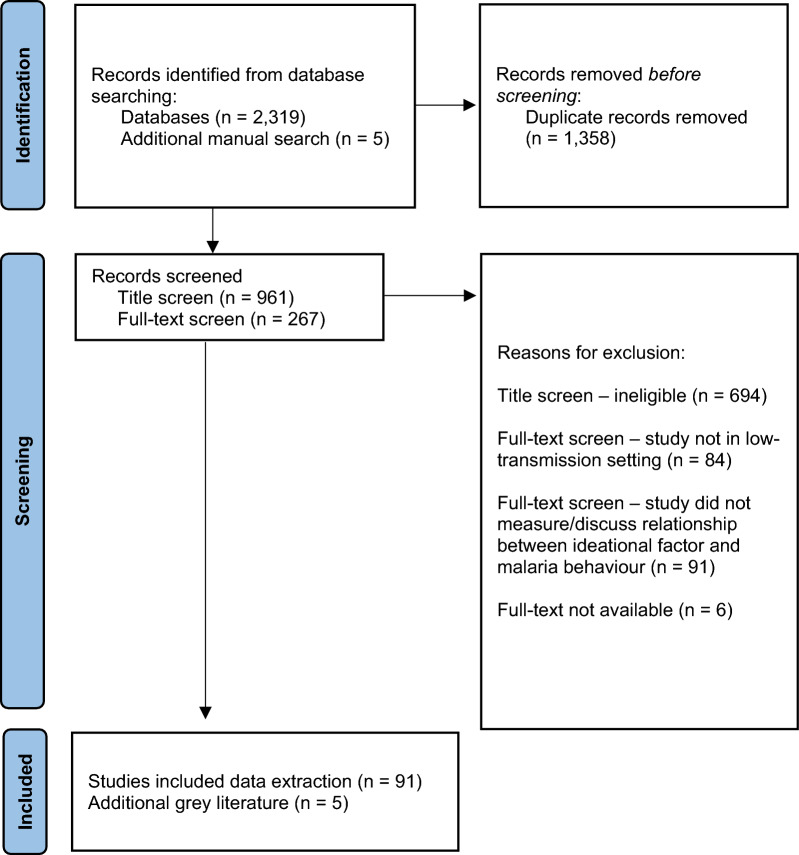
PRISMA diagram

A total of 961 titles were screened for inclusion, resulting in 267 publications. that qualified for further screening. Following title screening, a full-text screening was conducted to confirm that the publication met inclusion criteria, which included verification of API or *P. falciparum*/*P*. *vivax* prevalence data as available in the country of publication. The selection process aligned with PRISMA guidelines [26] (Fig. 2). During the full-text screening, 181 publications were determined to be ineligible. Common reasons for exclusion included: the study not taking place in a low-transmission setting (n = 84); the study not measuring or discussing the relationship between psychosocial ideational factors and a malaria behaviour (n = 91); or the full text not being available (n = 6). Five additional grey literature publications were purposively selected, based on collaboration with institutional partners. A recently completed systematic review [27] was not included in the study, but purposively reviewed to ensure that relevant studies were captured.

### Study location and population

The majority of research publications (58%) focused on an Asian country. The Greater Mekong Subregion (Cambodia, China (Yunnan Province), Laos, Myanmar, Thailand, and Vietnam) accounted for 25 publications (27%), while Bangladesh, India, Indonesia, Malaysia, and Sri Lanka were also represented. Twenty-nine publications (32%) focused on an African country, with Ethiopia (n = 10), Tanzania/Zanzibar (n = 4), and Zambia (n = 3) the most common locations of research. Countries representing South America, the Pacific, and the Middle East regions encompassed the remaining 10% of publications. Four studies included multiple countries.

Nineteen articles (20%) specified a focalized subpopulation as the subject of the research, citing an increased risk of transmission among the subject group in an otherwise low-transmission area. These subpopulations were identified largely (but not exclusively) based on occupation and included forest workers [28–30]; rubber tappers [31, 32]; construction workers [33]; military/security personnel [34]; seasonal migrant workers and miners [35–39]; subsistence agricultural workers [40, 41]; night-time venue workers [70]; refugee communities [42]; and internally displaced communities [43]. Often initial engagement through interviews and/or focus group discussions with key informants, such as Ministry of Health (MOH) staff, National Malaria Programme (NMP) staff, and/or local leaders not only helped to identify which subpopulations experience increased risk in low-transmission settings but also provided information on where and how to engage these populations in data collection.

The remaining articles (n = 72) collected data from general community members, health care providers, or caregivers in settings characterized by low transmission. Pregnant women, child caregivers, and households with children under 5 years old were the focus of only 12 studies. While these groups were not determined to be at additional risk of exposure relative to their communities, they are more likely to develop severe malaria disease than the general population [44]. Further, women of reproductive age living in low-transmission settings have lower acquired immunity to malaria than women living in high-transmission settings [44].

### Study design

Table 2 summarizes the study design of included articles, as well as the frequency of measurement for each psychosocial factor and type of malaria behaviour (i.e., prevention or treatment). A total of thirty-one studies (34%) included data on prevention behaviours only, while seven studies (8%) included data on treatment behaviours only. Forty-six studies (51%) included data on both preventive and treatment behaviours. Finally, five studies [34, 45–48] described psychosocial variables in a setting of low malaria transmission but did not measure associations with human behaviour. Common prevention behaviours measured include the use of ITNs, participation in IRS, and willingness to accept LSM in one’s community. Common treatment behaviours included willingness to accept MDA and seeking testing and care at a health facility.

**Table 2:** Summary of included articles: measures and study design

	Psychosocial and behavioral measures			Study design		
	Prevention behavior	Treatment behavior	Prevention and treatment behavior	Quantitative	Qualitative	Mixed-methods
Knowledge	[30, 31, 35, 37, 39, 40, 51, 53, 58–75]	[36, 42, 78–82]	[28, 29, 32, 33, 38, 41, 43, 49, 50, 52, 56, 80, 83–115]	[28, 30, 33, 34, 37–39, 41–44, 47, 49–51, 59–61, 63, 64, 68, 70, 71, 73–75, 78–80, 84, 87–103, 107–109, 111–114]	[29, 45, 52, 53, 56, 103]	[31, 32, 35, 36, 40, 46, 58, 62, 67, 69, 72, 81, 82, 85, 86, 105, 106, 110]
Attitude	[31, 39, 40, 44, 53–55, 58, 60, 63–68, 70–72, 74, 76, 77]	[36, 78, 81, 82]	[28, 32, 33, 43, 49, 50, 52, 56, 80, 89–92, 94–96, 100–106, 110, 112]	[28, 33, 39, 43, 49, 50, 60, 63–66, 68, 70, 71, 74, 78, 80, 89–92, 94–96, 100–103, 112]	[45, 48, 52–56, 104]	[31, 32, 35, 36, 40, 46, 58, 67, 72, 77, 81, 82, 105, 106, 110]
Perceived risk	[31, 35, 40, 53–55, 57, 60, 61, 66, 67, 76, 77]	[81, 82]	[29, 41, 52, 56, 80, 84, 91, 97, 101, 104, 106, 108, 110, 111]	[41, 60, 63, 66, 76, 80, 84, 91, 97, 101, 108, 111]	[29, 48, 52–56, 104]	[31, 35, 40, 67, 77, 81, 82, 106, 110]
Perceived response-efficacy	[35, 48, 53, 76]	None	[45, 102, 105]	[76, 102]	[45, 48, 53]	[35, 105]
Perceived self-efficacy	[30, 64, 76]	None	[56, 87]	[30, 64, 76, 87]	[56]	None
Descriptive norm	None	None	[104]	None	[104]	None
Subjective norm	[55]	None	None	None	[55]	None
Injunctive norm	None	None	[56]	None	[56]	None
Interpersonal communication	None	[42]	None	[42]	None	None

A quantitative design was used in 60 studies, 93% of which applied a one-time cross-sectional survey. Four studies [42, 49–51] used a pre-post longitudinal survey design. Ten studies [29, 45, 48, 52–57] implemented a design using only qualitative methods such as focus group discussions and in-depth interviews. The remaining studies utilized a mixed-methods approach, which most commonly included a combination of cross-sectional surveys and in-depth interviews, focus group discussions, or qualitative observations with a subset of the survey sample.

Sample designs included cluster random sampling (n = 28), stratified random sampling (n = 8), simple random sampling (n = 11), purposive sampling (n = 38), and convenience sampling (n = 5). Purposive methods were often employed using time-location and respondent-referral methods such as snowball and respondent-driven sampling, particularly when authors aimed to collect data from individuals whose work or social environment places them at increased risk of transmission. These methods may be useful among subpopulations that are highly mobile, and for whom it is difficult to establish a traditional sampling frame. However, while each of these approaches contains strengths, variable study designs and sampling strategies poses challenges when attempting to compare findings across settings.

### Psychosocial measures

Knowledge of malaria was the most commonly measured psychosocial ideational factor and was included in 82 publications (86%). This measurement often included quantitative questions prompting a participant to identify the cause of malaria and common symptoms of infection, ways to diagnose malaria, and ways to prevent infection.

Individual attitudes (59% of publications), and perceived risk of contracting malaria (36% of publications) were routinely measured and discussed. Attitudes were measured in multiple ways, and generally referred to one’s perception related to a care-seeking or prevention measure, e.g., agreement that sleeping under a bed net is comfortable; insecticide-treated clothing is durable or pleasant to wear [31], that the benefits of spraying interior walls outweigh the risks [65]; there is no alternative to seeking care at a health facility when a child has fever; and that care-seeking should occur within 24 h of the onset of fever [103].

Qualitative measures of attitudes primarily utilized open-ended questions to focus on reasons why an intervention is not considered acceptable [29, 48, 65, 104]. Quantitative attitudinal measures were often limited to agreement with one or two statements. While a different psychosocial construct, measures of perceived risk were often miscategorized as “attitudes” in studies and included agreement with closed-ended statements that malaria is dangerous, can be deadly, and/or that the respondent is vulnerable to malaria [53–55, 66, 91], although several studies assessed perceived risk via open-ended interview questions [29, 76, 84].

Other psychosocial factors including perceived response efficacy (i.e., one’s belief that a certain action and/or commodity will avoid or resolve the problem; 9% of publications), perceived self-efficacy (i.e., one’s confidence in their ability to perform a specific behaviour; 6% of publications), and interpersonal communication (1% of publications) were less commonly measured by the literature.

### Linkages between psychosocial factors and malaria behaviours

A review of these 91 studies offers evidence for multiple linkages between psychosocial factors and malaria behaviours. Prevention and treatment-seeking behaviours were routinely measured across the reviewed articles. Prevention behaviours included net use, net procurement, net maintenance, acceptance of IRS, acceptance of SMC, and smaller-scale interventions such as the use of treated clothing. Treatment behaviours included acceptance of MDA, seeking care for malaria symptoms, types of treatment services sought (e.g., hospital-based services or traditional medicine services), adherence to treatment, and factors motivating prompt or delayed treatment seeking.

Among articles that measured knowledge, there were generally positive associations between one’s level of knowledge about malaria (symptoms and causes) and their behaviour. However, studies also largely cited a high rate of correct knowledge at the time of study while rates of malaria-related behaviours varied. This suggests that knowledge alone may not be sufficient to achieve behaviour change (for examples, see [53, 81–83]). While the majority of studies focused on community member knowledge as the subject, others such as Hein et al*.* [36] assessed health care provider knowledge and discussed a positive relationship between health care provider knowledge of malaria and testing patients with fever for malaria.

Evidence from the reviewed studies suggests that individuals with a high level of perceived susceptibility to malaria are more likely to practice preventive or treatment behaviours [53, 54, 81, 82]. Studies focusing on child caregivers also frequently measured this variable with questions assessing the caretaker’s perception of whether they or the child were at risk for severe malaria. This data was collected in combination with prevention behaviour data. Perceived susceptibility and severity were commonly reported as low in areas of low transmission [41, 60, 61, 76, 84, 101, 108, 111]. Authors in these studies discuss that this may be because the individual does not consider malaria to be a common problem in their community.

Perceived response efficacy of recommended prevention and/or treatment behaviours was measured in seven studies [35, 45, 48, 53, 76, 102, 105], using both quantitative (Likert-scale) and qualitative measures. Of these articles, three assessed the acceptability and perceived effectiveness of ITN usage, two assessed the perceived effectiveness of IRS, one assessed the perceived effectiveness of healthcare worker efforts to increase malaria prevention behaviours, and one assessed the perceived effectiveness of MDA. Results indicate inconsistent associations. A study on perceived effectiveness and MDA participation [34] yielded positive associations, as did a study assessing acceptance of IRS [48]. Other studies, however [45, 76, 102, 105], did not yield a positive association between perceived response efficacy and malaria behaviour. This may be due to other factors producing a stronger effect on the behaviour, such as perceived risk [53].

Other psychosocial determinants, including perceived self-efficacy, interpersonal communication, and norms (i.e., subjunctive, injunctive, and descriptive norms) were not consistently measured across studies. Thus, the study team did not conduct an assessment to identify trends in their relationship to behavioural outcomes.

## Discussion

There are several implications of this review for future malaria research and practice in settings of low transmission. These have been summarized in brief, and are presented in Table 3. Each of these implications are then described in further detail.

**Table 3:** Summary of findings and potential actions for Malaria control programmes

Finding from review	Potential actions for Malaria control programme
1. Limited measurement of psychosocial factors Few studies measured psychosocial factors beyond knowledge and attitudes. Where factors other than knowledge and attitudes were measured, there was often an association found with malaria behaviors	• Enhance research tools to include a broader range of psychosocial factors (e.g., norms, self-efficacy, interpersonal communication) in studies and surveys
2. Positive association of knowledge, perceived risk, and behavior Studies consistently found positive associations between knowledge, perceived risk. and malaria preventive behaviors	• Strengthen community engagement programmes to reinforce the seriousness of malaria and its continued threat in low-transmission context, particularly among vulnerable groups
3. Lack of consistency in measurement There is generally a lack of consistency and standardization in measuring psychosocial variables across studies	• When appropriate for the study context, advocate for the use of standardized measures. Programmes should utilize both quantitative and qualitative approaches. Prior to survey data collection, programmes should verify through pre-test that the variables used to measure the construct are appropriate for the context
4. Limited focus on high-risk subpopulations Few studies addressed high-risk subpopulations like forest goers, miners, and night-time workers. Behavior change among these groups may require a different approach than the general population	• Expand research on high-risk subpopulations. Research may benefit from respondent-driven and time-location sampling techniques • Identify communication channels and optimal broadcast periods for high-risk subpopulations

While the review identified many studies investigating psychosocial drivers of malaria-related behaviours in low-transmission contexts, few included a full range of psychosocial factors that are known precursors to behaviour change. Knowledge, attitudes, and perceived risk regarding malaria were the most common factors measured in the reviewed studies, while other psychosocial factors were neither commonly nor sufficiently measured. These poorly studied factors include descriptive and injunctive norms, perceived response efficacy, perceived self-efficacy, and interpersonal communication. Each of these variables has been shown to influence malaria behaviours in high-transmission settings, and future research in low-transmission settings would benefit from incorporating these variables into research tools.

Studies that measured knowledge and/or perceived risk of malaria generally yielded positive associations with malaria preventive behaviour; for instance, an individual’s level of knowledge or perceived risk was routinely found to be positively associated with their practice of a malaria preventive behaviour in the studies reviewed. In Southeast Asian contexts, these results align with those found in a recent systematic review by Cheng et al. [119], which suggest knowledge of malaria, perceived response efficacy, and positive attitudes toward ITNs are key factors associated with their use. Similarly, positive attitudes towards MDA programmes, perceived self-efficacy, and joint decision-making were emphasized as important influencing factors of MDA participation. Rates of correct knowledge appear to generally be high among the populations studied in the Cheng et al*.* review, and thus do not offer much room for improvement by way of social and behaviour change programmes aside from maintaining current knowledge. In the present review, individuals with high perceived risk (susceptibility and severity) toward malaria were more likely than those without this perception to practice preventive or care-seeking behaviours. However, perceived risk may tend to remain low in areas that are not characterized by high transmission, as the individual may not consider malaria to be a common problem in their community [40, 55]. Malaria SBC programmes in low-transmission settings may see improved outcomes from reinforcing that malaria is serious and continues to pose a threat to the community, particularly among groups subject to increased risk of exposure or vulnerability.

To determine the links between psychosocial factors and malaria behaviours across settings and over time, additional work is needed to clearly define and standardize measurement. Few reviewed studies included an element of theory, the importance of which has recently been discussed in the context of malaria control [120]. Using theoretical models to hypothesize the relationships between psychosocial factors and behaviour will improve the research design, quality of the research, and local relevance of findings. In the current review, psychosocial variables were often defined and measured differently across studies. Indeed, there is not currently a consensus or a shared definition of most psychosocial variables, nor is measurement consistent across settings. This can be achieved through utilizing standard measures such as those included in questionnaires for the Malaria Behavior Survey (MBS), the Malaria Indicator Survey SBCC module, and the RBM Partnership to End Malaria SBC Indicator Reference Guide. Consistent collection of this set of variables across a range of settings allows for comparison across contexts and over time, while providing the context- specific information needed to inform malaria programmes [120]. Given the positive associations found between perceived susceptibility or severity and practicing malaria behaviours, it will be important for researchers to measure both, as well as behaviours, in settings of low transmission.

This challenge in measurement presents an opportunity for future research in low-transmission settings. In particular, the MBS has been implemented in several high-transmission countries. MBS instruments were designed in collaboration with the RBM partnership and survey items validated in several countries. The MBS recently launched a version of the questionnaire tailored to low-transmission settings. Future research seeking to measure multiple psychosocial variables and malaria behaviours may benefit from using MBS instruments as a point of departure. Broad application of this research method may produce more consistent and comparable measurement of psychosocial determinants, particularly malaria attitudes, which were inconsistently measured among the reviewed studies. Data may then be integrated into ongoing SBC interventions to ensure efficient and strategic use of resources in addressing the determinants that shape malaria prevention and treatment behaviours in low-transmission settings.

There are few published articles focusing on subpopulations that experience disproportionate risk of exposure such as forest goers, gold (or other mineral) miners, rubber tappers, or night-time workers, although there is some recently emerging research to this effect [121]. Studies that focus on these subpopulations utilize respondent-driven sampling and time-location sampling techniques. These studies also routinely engaged government personnel, such as MOH and NMP staff, as well as community leaders before determining an appropriate sampling strategy. Formative engagement such as this has been described in detail and is recommended by the UCSF High Risk Populations Surveillance and Response Guide [122]. As malaria elimination activities may focus further on these subpopulations, there is opportunity and a need to strengthen the sampling methods, as well as the methods to assess and quantify the psychosocial variables that influence malaria prevention and treatment behaviours in low transmission settings. Future research should maintain the practice of engaging with key stakeholders early in the research process to ensure the population sampled is relevant and that research findings can be translated into programmatic applications.

There are several limitations to this review. First, only English publications were included, which may limit the inclusion of technical reports and publications in other languages. This may particularly limit the inclusion of materials from Francophone African contexts such as Senegal. Second, the psychosocial variables included in key search terms drew from the ideation model. While this is not the only model that includes psychosocial determinants of behaviour, it draws on constructs from multiple behavioural theories, including the Health Belief Model, the Theory of Planned Behaviour, the Extended Parallel Process Model and the Social Cognitive Theory and, therefore, is expected to cover a broad range of relevant factors. The authors feel that, overall, this article provides a useful synthesis of available evidence on psychosocial drivers of malaria in low transmission settings. Settings such as South America were more limited than expected. This appears due to the screening criteria. Often, studies based in Central and South America, and the Caribbean measured an ideational factor and behaviour, but did not test the factor-behaviour association, thus rendering the article ineligible. Finally, findings of this review are not intended to suggest that addressing psychosocial determinants alone are sufficient to change behaviour. In the context of malaria elimination, behaviour change relies on functioning health systems, including strong malaria elimination and control programmes. Indeed, broad investments in health systems are requisite to achieve elimination of malaria, as has been previously advocated for by academics and the WHO [116–118].

## Conclusions

The review highlights the need for more consistent, comprehensive measures of psyctors and the importance of reaching subpopulations at higher risk of transmission in low transmission contexts. Malaria-related knowledge is generally high, even in settings of low transmission. However, malaria-related knowledge in these settings is lower among sub-populations who experience the highest malaria risk. Programmes and research aiming to improve malaria-related behaviours in these settings should work to better understand the psychosocial factors that have been positively associated with prevention and care-seeking behaviours, such as norms, perceived response efficacy, perceived self-efficacy, and interpersonal communication. While these factors are not necessarily distinct from psychosocial factors identified in similar research focused in high malaria transmission settings, it is important to better understand how each factor may influence behaviour in a low transmission context. Further, the implications for malaria behaviour change programming are different and an area in need of further research. Existing research tools and approaches are available to support more systematic collection of psychosocial determinants and improved sampling approaches and should be applied more widely. In low-transmission settings, these tools can provide useful information on people’s beliefs and perceptions toward local interventions. As interventions in low transmission settings are often tailored, capturing this type of data may be useful for both programme evaluation and quality improvement.

### Supplementary Information

**Additional file 1. **Search terms list.

**Additional file 2:** Literature table.

## Data Availability

Key search terms used to execute the search are available in Additional file 1. The charted results of the review are available in Additional file 2. As this is a review article, no other datasets were generated or analyzed.
