# Supplementary material for: Understanding psychosocial determinants of malaria behaviours in low-transmission settings: a scoping review
Source: Malar J. 2024 Jan 10;23:15. doi: 10.1186/s12936-023-04831-9 (PMC10782749; doi:10.1186/s12936-023-04831-9)
Supplement: Supplementary file 1 — Additional file 1. Search terms list. [file 12936_2023_4831_MOESM1_ESM.docx]

Additional File 1: Search Terms List

| **List of Key Search Terms** | | |
| --- | --- | --- |
| *Malaria* | | |
| **AND** | | |
| 1 or more of the following ideational factors:  *Knowledge; Attitudes; Perceived Severity; Perceived Vulnerability; Perceived Susceptibility; Perceived Self-efficacy; Perceived Self efficacy; Interpersonal Communication; Personal Advocacy; Decision-making Autonomy; Agency; Ideation; Ideational; Myths; Beliefs; Norms;* | | |
| **AND EITHER** | | |
| 1 or more of the key geographic terms:  *Low-transmission area*  *Low-transmission zone*  *Low-transmission country*  *Low-transmission setting*  *Elimination* | **OR** | 1 or more of the key subpopulations:  *Seasonal Workers*  *Migrant Workers*  *Men*  *At-risk groups*  *High- risk groups*  *At risk groups*  *High risk groups*  *Internally Displaced Persons*  *IDPs* |
